# *Benincasa hispida* Alleviates Stress and Anxiety in a Zebrafish (*Danio rerio*) Model

**DOI:** 10.3390/life14030379

**Published:** 2024-03-13

**Authors:** Nityashree Kyathegowdanadoddi Lakshmanagowda, Niju Sagar, Rachitha Puttasiddaiah, Kandi Sridhar, Vinay Basavegowda Raghavendra, Maharshi Bhaswant

**Affiliations:** 1 Department of Clinical Psychology, JSS Medical College and Hospital, Mysuru 570015, India; nityashree.30@gmail.com; 2 Teresian College Research Center, Teresian College, Mysore 570011, India; nijusagar2204@gmail.com (N.S.); biotechrachitha@gmail.com (R.P.); 3 Department of Food Technology, Karpagam Academy of Higher Education (Deemed to be University), Coimbatore 641021, India; sridhar4647@gmail.com; 4 New Industry Creation Hatchery Center, Tohoku University, Sendai 9808579, Japan; 5 Center for Molecular and Nanomedical Sciences, Sathyabama Institute of Science and Technology, Chennai 600119, India

**Keywords:** antistress, antianxiety, *Benincasa hispida*, behavioral parameters, therapeutic molecule

## Abstract

The Ayurvedic medical system uses fruits of the *Benincasa hispida* plant to treat mental diseases, including schizophrenia. The goal of the current study was to assess the aqueous extract of *B. hispida* fruit’s ability to relieve stress and anxiety induced in zebrafish models using neuropharmacological evaluation, which included determining behavioral parameters in tests such as the T-maze, open tank test (OTT), and light–dark preference test (LDPT). After measuring the zebrafish survival rate for 96 h, the LC_50_ was found to be 5 µg. AChE (acetylcholinesterase) inhibitory activity and the status of antioxidant enzymes (SOD, CAT, and LDH) were also used to evaluate the toxicity. Furthermore, the administration of the aqueous extract of *B. hispida* fruit increased the frequency of entry and duration of time spent in the bright section, suggesting a noteworthy reduction in levels of stress and anxiety. Additionally, the antistress and antianxiety activity was confirmed by the docking studies’ mechanism of action, which involves the AChE receptor binding stability of the homogalactaconan molecule found in the aqueous extract of *B. hispida* fruit. Overall, the findings of this study demonstrated that the aqueous extract of *B. hispida* fruit is a viable therapeutic molecule for the creation of novel drugs and the treatment of stress since it has the therapeutic advantage of reversing the negative effects of stress and anxiety.

## 1. Introduction

Traditional medicine is a holistic approach rooted in indigenous theories, beliefs, and experiences that involve the maintenance, prevention, diagnosis, improvement, or treatment of physical and mental illnesses [1]. The potential of natural ingredients has been recognized as a method for creating antistress and antianxiety drugs [2]. Accurate identification of medicinal plants is crucial since the true nature of the raw material determines the medicine’s effectiveness [3]. The use of plant parts, such as stems, roots, leaves, fruits, and flowers, as a source of drugs for a range of human illnesses has drawn growing attention from researchers [4]. The annual plant *B. hispida* is used as a vegetable on hills up to 1200 feet in elevation and grows abundantly across India’s plains [5]. *B. hispida*, often known as a wax gourd, is an essential ingredient in kushmanda lehyam, an Ayurvedic rejuvenator used to treat epilepsy and other neurological disorders. The Ayurvedic medical system treats schizophrenia and other mental diseases with the fruits of *B. hispida* [6,7]. The fruit is a rich source of antioxidants, including carotenes, flavonoids, and sterols, which help combat oxidative stress and provide essential vitamins, minerals, and volatile oils making it effective in combating oxidative stress [8].

Stress is a nonspecific body response to demanding situations, characterized by anxiety or mental tension. It is a normal human reaction, pushing us to deal with problems and dangers. However, how we handle stress significantly impacts our overall feelings [9]. Conventional definitions of stress include a stimulus, or “stressor”, that the person encounters, such as a job loss a laboratory shock, or a reaction characterized by unpleasant feelings, especially anxiety, and physiological arousal. Individuals who experience dysregulation of their stress circuits are more susceptible to major psychiatric problems such as depression, occupational stress disorder, and post-traumatic stress disorder [10,11]. The literature provides a no precise definition of anxiousness. Even if the distinguishing characteristics are distinct and easily identified. The American Psychiatric Association defines anxiety as the expectation of a threat or adverse event, followed by feelings of dysphoria or physical symptoms connected to stress. The variables that pose a threat might originate from both the external and internal domains. It seems that anxiety is a distinguishing feature of contemporary life and is almost a defining cliché [12]. For a very long time, zebrafish (*Danio rerio*) have been utilized as animal models in biomedical research, particularly in studies about development and genetics. Because the zebrafish is a fairly basic vertebrate animal that shares many physiological characteristics with humans, scientists may use it to study the pathways and processes linked to the genesis of human disease and medical interventions. Zebrafish include all ‘classical’ vertebrate neurotransmitters. Their neuroendocrine system also generates strong physiological responses to stress. Zebrafish are also an excellent choice for scientific research since they are inexpensive, low-maintenance, and have high numbers of progeny [13,14,15].

The purpose of this study was to assess the anxiolytic qualities of the aqueous extract of *B. hispida* fruit using the open tank test, T-maze, and light–dark test in zebrafish, taking into account its pharmacological characteristics. Furthermore, to support the traditional usage of this medicinal plant for treating anxiety and stress problems, we evaluated stress using the same model organism [16]. To the best of our knowledge, no prior study has examined the anxiolytic properties of the aqueous extract of *B. hispida* fruit and similar compounds using zebrafish. Our goal is to contribute experimental evidence to this field’s corpus of knowledge. Here, we assessed how the aqueous extract of *B. hispida* fruit affected the behavioral traits of zebrafish associated with stress and anxiety.

## 2. Materials and Methods

### 2.1. Preparation of Aqueous Extract of B. hispida Fruit

The aqueous extract of *Benincasa hispida* fruit was prepared by a simple maceration process. The peel of *B. hispida* was washed with tap water followed by distilled water until all the impurities were removed. At that time, it weighed about 8 kg. The fruit was cut into pieces and the seeds were removed and kept separately. The pulp was mashed and finely grated to afford a soft mass and later on it was spread on a cloth under shade to dry. It was shade-dried for about 5 days.

### 2.2. Phytochemical Assay of Aqueous Extract of B. hispida Fruit Pulp

#### 2.2.1. Phytosterols—Salkowski Reaction

H_2_SO_4_ (1 mL) was added from the test tube’s sidewalls to 0.5 mL of extract in each test tube. The presence of phytosterols is indicated by the color’s reddish-brown appearance [17,18].

#### 2.2.2. Saponins—Foam Test

The extract was added to a small amount of water in a test tube, and the tube was vigorously shaken. The appearance of foam and its ten-minute persistence point to the presence of saponins [19].

#### 2.2.3. Alkaloids—Dragendroff’s Test

A few drops of Dragendroff’s reagent (potassium bismuth iodide) were added after the extract had been dissolved in methanol and had become acidic. A precipitate that is orange or red in color suggests the presence of alkaloids [20].

#### 2.2.4. Carbohydrates—Fehling’s Test

Fehling’s A and B solution was used to warm the extract. The precipitate’s orange–red appearance indicates the existence of carbohydrates [21].

#### 2.2.5. Anthraquinone—Anthraquinones Test

The diluted ammonia (1 mL) was added after 5 mL of extract had been hydrolyzed with H_2_SO_4_ and extracted with benzene. The presence of anthraquinones is indicated by a rose–pink color [22].

#### 2.2.6. Flavonoids-Ferric Chloride Test

When a few drops of a neutral ferric chloride solution are added to the alcoholic solution of the plant extract, a green colouring develops, indicating the presence of flavonoids [23].

#### 2.2.7. Phenolic Compounds and Tannins—Ferric Chloride Test

A test tube containing 2 mL of extract received drops at a time of ferric chloride solution. The presence of tannins and phenolic compounds is indicated by the presence of a bluish-black precipitate [24].

#### 2.2.8. Steroids—Steroids Test

The methanol (10 mL) was used to dissolve 1 mL of the extract, and an equivalent volume of concentrated H_2_SO_4_ was then added. The presence of steroids is indicated by the upper layer’s red coloring and the sulfuric acid layer’s yellow with green fluorescence [25].

#### 2.2.9. Proteins—Biuret Test

To the Biuret reagent 2 mL of extract that had been dissolved in methanol was added. The contents were thoroughly shaken and given a water bath warming. Red or violet coloration indicates the presence of proteins [26].

#### 2.2.10. Fixed Oils and Fatty Acid—Spot Test

On the filter paper, a spot was formed along the plant extract and dissolved in methanol. The presence of fixed oils and fats is indicated by the appearance of oil stains on the filter paper [27].

### 2.3. Antioxidative Properties of B. hispida

The hydrogen peroxide scavenging assay (H_2_O_2_), nitric oxide (NO) scavenging, Polyphenol content, and 2,2-diphenyl-1-picrylhydrazyl (DPPH) were used to test the antioxidant activity of *B. hispida* [28,29].

#### 2.3.1. The 2,2-diphenyl-1-picrylhydrazyl Radical (DPPH) Assay

The effects of the *B. hispida* extract were predicted using a slightly altered procedure [30]. The DPPH (1 mM, SRL, Bangalore, India 99%) solution was dissolved in 100 mL of ethanol and then maintained at 20 °C. The ability of the *B. hispida* extract to scavenge free radicals was evaluated at various concentrations using Butylated Hydroxyl Toluene (BHT), (Himedia, Bangalore, India 98%) as the reference substance (1 mg/mL). The test samples were added to 1 mL of the ready-to-use DPPH solution and completely mixed with a vortex mixture before being incubated for 30 min in the dark. The absorbance was calculated at 517 nm. The inhibitory activity was determined according to Equation (1).

(1) $$Scavenging\left(\%\right)=\frac{OC-OS}{OC}\times 100$$

where: OC is the absorbance of the control (all the reagents except the test sample) and OS is the absorbance of the standard sample (BHT).

#### 2.3.2. Assay for Scavenging Hydrogen Peroxide (H_2_O_2_)

The hydrogen peroxide activity was evaluated, with a small adjustment, according to [31]. The mixture of the various concentrations of *B. hispida*, 200 mM sodium phosphate buffer, 10 mM deoxyribose, 10 mM EDTA (ethylenediaminetetraacetic acid), 10 mM H_2_O_2_, and deionized water was kept in the virtual incubator for 4 h. To stop the reaction, trichloroacetic acid (TCA) (2.8%) and thiobarbituric acid (TBA) (1% in 50 mM NaOH) were introduced. The solution was then boiled for 10 min, after which it was cooled with tap water. At 520 nm, the absorbance was measured. Methanol served as the blank and Ascorbic acid (A A) served as the standard. At 610 nm, the drop in absorbance was measured, and the percentage of H_2_O_2_ scavenging activity was determined according to Equation (2).

(2) $$Scavenging\left(\%\right)=\frac{HC-HS}{HC}\times 100$$

where: HC is the absorbance of the control (all the reagents except the test sample) and HS is the absorbance of the test sample (*B. hispida* and ascorbic acid).

#### 2.3.3. Scavenging of Nitric Oxide (NO) Radicals

When sodium nitroprusside interacts with physiological oxygen pH, nitric oxide radicals are produced. Different doses of aqueous extract of the *B. hispida* fruit were separately added to 100 µL of 5 mM sodium nitroprusside (Fischer Scientific, Bangalore India 9%), which had been measured at 30 min intervals by mixing with Griess reagent after producing up to 1 mL of reaction volume. The use of BHT as a positive control was contrasted with the scavenging of nitric oxide radicals [32]. At 550 nm, the absorbance started to decrease and the % decrease was determined according to Equation (3).

(3) $$Nitric\;oxide\;radical\;scavenging\;activity\;\left(\%\right)=\left[\left(\frac{A_{0}-A_{1}}{A_{0}}\right)\times 100\right]$$

where: A_0_ is the absorbance of the control (all the reagents except the test sample) and A_1_ is the absorbance of the test sample (*B. hispida*/BHT).

#### 2.3.4. SOD and Catalase Activities

Zebrafish brain samples were homogenized with a glass homogenizer to liberate cellular contents and produce a homogenous solution. After homogenization, the homogenate was centrifuged. Centrifugation at 4 °C for 10 min at 3000× *g* ensures the stability of delicate biological molecules and structures. The resulting supernatant was used for biochemical experiments such as SOD and catalase [33]. The methods of Elstner and Heupel (1976) and Aebi 1984 [34,35] were followed, respectively, to assess the superoxide dismutase activity and catalase activity. A UV/Vis Power Wave XS2 microplate reader (BioTek Instruments Inc., Winooski, VT, USA) was used for all measurements. For SOD activity at 37 °C, the wavelength was 450 nm, while for CAT activity, it was 405 nm [36].

#### 2.3.5. Acetylcholine Esterase

AChE activity was measured in the supernatant of each adult zebrafish brain homogenate for each treatment group, using a previously reported method. Padilla et al. [37] discovered that each zebrafish’s brain was segregated after being exposed to stress and anxiety. The tissues were homogenized in 500 μL of ice-cold sodium phosphate buffer (pH 8.0, 0.1 M) with 1% Triton-X 100 The samples were homogenized and centrifuged at 4 °C for 10 min at 3000× *g* to separate the supernatant. The resultant supernatant was then used to test acetylcholinesterase (AChE) activity, as described in the established procedure. This enzyme activity test is used to assess cholinergic function and can provide information about how stress and anxiety affect zebrafish brain activity. The supernatant was collected at each stage of the two centrifugations of homogenates (the first at 6000× *g* for 8 min and the second at 12,000× *g* for 10 min at 4 °C). 5,5′-dithiol bis-2-nitrobenzoic acid (DTNB) and acetylthiocholine iodide (ATCI) were used as test reagents in 96-well plates to assess AChE activity [38].

### 2.4. Behavioral Study

#### 2.4.1. Maintenance of Zebrafish

A total of 170 Zebrafish (*Danio rerio*) (wild-type strain; 4–6 months old) were maintained in glass tanks (dimensions: L × W × H = 100 × 45 × 45 cm) (100 L). The animals were obtained from a local commercial supplier (Sea World Aquarium, Mysuru, India) and were acclimated for at least 2 weeks before the experiments. A tank containing normal water was de-chlorinated and kept under constant aeration and mechanical, and chemical filtration (25–27 °C; pH 7–8; total ammonia at <0.01 mg L^−1^), and a 14:10 h light–dark cycle was provided by fluorescent lamp tubes (12 watts; lights on at 7:00 am). The chemical conditions of water were controlled by using commercial kits (Agappe, Mysuru, India) (pH, nitrate, and ammonia). Only animals weighing 450 mg and above were used. All animals were fed two times a day with Optimum micro pallet fish food. All procedures were performed according to the CPASEA guidelines, India (Committee for the Purpose of Control and Supervision of Experiments on Animals), And previously approved by the Animal Research Ethics Committee from JSSAHER (project proposal No. JSSAHER/CPT/IAEC/146/2023).

#### 2.4.2. Animal and Treatment

Once the 5 µg/mL LC_50_ was determined, a safe dosage was found to be between 1 µg and 3 µg/mL. The entire study was based on three primary parameters: toxicity (10 fish per group in five separate groups, including the control, 2.5 µg/mL, 5 µg/mL, 7.5 µg/mL, and 10 µg/mL). Ten fish divided into five groups—Control, Stress-Induced, Standard, Low, High, and Environmental—were stressed. and anxious (ten fish divided into five groups: environmental, low, high, control, anxiety-induced, and standard). Following the treatment conditions, three fish were removed from each group for behavioral study, three fish for biochemical estimation, and three fish for environmental investigation.

#### 2.4.3. LC_50_

The aqueous extract of the *B. hispida* fruit was tested for its toxicity to zebrafish. On the 1st day, healthy zebrafish were chosen randomly from the fish tank, weighed (more than 450 mg), and transferred into 5 separate labeled bowls (Micro oven plastic bowls can hold 1.5 L of water). Each bowl of fish (10 fish in each bowl) was labeled as either control, 2.5 µg/mL dose (aqueous extract of *B. hispida* fruit), 5 µg/mL dose, 7.5 µg/mL dose, and 10 µg/mL dose. Different doses were prepared from the aqueous extract of *B. hispida* fruit that was used to determine the toxicity to Zebrafish.

#### 2.4.4. Stress Protocol

Fish that weighed more than 450 mg were chosen for the experiment. The experimental design was planned for 6 days, wherein for 4 days we induced different stressors at different times, later the treatment period was given by dividing the fish into 4 different groups: standard, low dose, high dose, and environmental (10 fish in each group). Four bowls were taken, and they were labeled as control, standard, low (1 µg), and high (3 µg). In each bowl, there were 7 fish. The fish were submitted twice a day to one of the following stressors for 4 days: chasing—wherein fish were chased using a glass rod stirrer manually. Cooling—the normal water was cooled to 20 °C in a bowl and the fish were left for 5 min for 3 trials each with an interval of 2 min. Social Isolation—each fish was separated into beakers for 3 h. Crowding—all the fish were added to one single beaker wherein there was no sufficient space to swim. Restrained stress—an Eppendorf of 2 mL was holed in such a way that water flowed from both sides, each fish was kept inside the Eppendorf and left inside the water bowl for 1 h. Low water level—this was similar to the crowding test but a little more water was provided so that the fish could swim, but not very freely. Net stress—each fish was lifted out of the water using a net for 60 s. Tank water replacement—tank water was frequently changed every 10 min for 1 h (Table 1).

The fish were injected with an aqueous extract of *B. hispida* fruit into the intra-peritoneal region on the fish, by anesthetizing the fish through cold water at 10–15 °C on the same day. One fish from each group was tested for its behavior using the dark and light test, open-field test, and T-maze test after four days of stress. Diazepam was weighed at 5 µg and dissolved in 1L of water for the standard group to compare the effect of treatment with the standard and aqueous extract of *B. hispida* fruit treatment on stress, while the control group was kept unaltered. Three fish were euthanized through cold water at 2 °C and their brains were extracted via dissection [39].

#### 2.4.5. Anxiety Protocol

The anxiety study design was kept for one day wherein caffeine was used as a drug to induce anxiety by exposing the fish for about 15 min to caffeine which was mixed with 1 L of water in a bowl. Later we divided the fish into 4 groups: standard, low dose, high dose, and environmental for treatment using the aqueous extract of *B. hispida* fruit. Fish that weighed more than 450 mg were chosen so that the fish could sustain the caffeine doses. The concentration of caffeine in the tank water was 100 mg/L. Ten fish from each group were anesthetized and intraperitoneally injected with an aqueous extract of *B. hispida* fruit. The control group received an injection with saline solution, while the standard group received 5 µg/mL of diazepam, and the low dose and high dose groups received 1, and 3 µg/L of aqueous extract of *B. hispida* fruit, respectively. Three fish were euthanized after 24 h by injection [40].

#### 2.4.6. Intraperitoneal Injection

The fish were immersed in cold water at 10–15 °C for 30–60 s to anesthetize them, or until there was only gill movement in the fish. When the fish cannot move, tap on the beaker to make sure the fish is anesthetized. Transfer the fish to a watered sponge holder to inject the fish into its intraperitoneal region. Since this is a time-sensitive procedure, all the steps have to be completed within 30–40 s after anesthetizing the fish. Transfer the fish to a beaker that holds three to four liters of water so that the fish can recover from the cold-water anesthesia [41].

#### 2.4.7. Open-Field Test

The open-field test is a frequently used method of assessing zebrafish behavior and locomotion. This was chosen as an adaptation of the designs which also utilized top-down measurement of horizontal fish movement. After each trial, the water was replaced since, substances released by a fish can influence the behavior of the fish used in the next trial, invalidating the result. Water temperature was maintained at between 25 and 28 °C for all trials. Zebrafish were assessed across virtual zones in a testing arena in a tank with a height of 15 cm. The tank was filled to a depth of 6 cm with normal water which was changed every trial [42].

#### 2.4.8. Light–Dark Test

The zebrafish were subjected to light and dark tests for 5 min. The amount of time spent in each zone (dark and light) and the number of entries to each zone were considered behavioral parameters. A quick back into the dark container after making a partial entry into the white compartment was the definition of risk assessment behavior. The device was a tank that measured 15 × 10 × 45 cm in height, depth, and length. It was evenly divided into two separate compartments, one coated in a white material and the other in a black sheet of thick paper, where the light rays could not enter the tank. From one side of the tank, the light was on so that only half of the tank could receive light, this test was conducted in a dark room [43].

#### 2.4.9. T-Maze Experiment

With a few minor adjustments, we employed the previously reported methodology for learning and memory [44]. Every experiment was conducted for 6 h for 3 trials of 5 min each with a 2 min break at each trial. There was one stem and two arms wherein one was colored red and the other was colored green, which made up the T-maze. For four days in a row, the zebrafish were trained with one trial per day. The green-colored arm section held 20 µg of food, which was brine shrimp, throughout the training sessions, and the red-colored arm contained frequent manual chasing behavior (which creates fear in the fish) with a glass rod by the experimenter. Based on these trials the fish tries to assume that the red arm is harmful and the green arm is safe (cognitive map). Following a 4-day training session, memory testing was conducted on all experimental zebrafish on the fifth day.

#### 2.4.10. Environmental Enrichment

This was an environment created in an aquarium that had similar dimensions to the original tank and that was filled with natural sand, live plants, stones, and pebbles to enrich the environment for the fish. So that we can clearly understand the effect of treatment with an aqueous extract of *B. hispida* fruit and the effect of the natural environment on fish.

#### 2.4.11. Statistical Analysis

The data were presented as means with standard deviation. The data were assessed for normality using the D’Agostino–Pearson omnibus normality test. The data were analyzed using one-way ANOVA and Duncan’s test. *p* ≤ 0.05 was considered significant (* 0.01, ** 0.001, *** 0.0001) using SPSS and MS Excel.

## 3. Results

### 3.1. Preparation of Aqueous Extract of B. hispida Fruit

**Figure 1:**
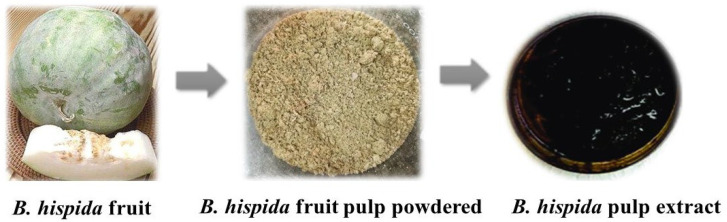
Preparation of aqueous extract of *B. hispida* fruit.

After the fruit sample was extracted, the yield percentage of the aqueous extract of *B. hispida* fruit was found to be 4.84% (Figure 1).

### 3.2. Phytochemicals Analysis

Phytochemical screening of the *B. hispida* fruit in an aqueous extract shows the presence of phytosterols, saponins, anthroquinone, phenolic compounds, and steroids (Table 2).

### 3.3. Antioxidant Assay

#### 3.3.1. DPPH (2,2-diphenylpicrylhydrazyl) Radical Assay

The aqueous extract of *B. hispida* fruit inhibited DPPH activity showing 54.56 ± 1.08 µg/mL scavenging activity compared to standard (BHT) at 40.41 ± 1.6 µg/mL (Table 3). The color changes likely resulted from H+ atom transfer to DPPH stable molecules.

#### 3.3.2. H_2_O_2_ Radical Scavenging Activity

The aqueous extract of *B. hispida* fruit showed a high H_2_O_2_ radical scavenging activity, with an IC_50_ of 67.53 ± 0.05 µg/mL, comparable to standard ascorbic acid 44.37 ± 1.9 µg/mL (Table 3).

#### 3.3.3. Nitric Oxide Radical Scavenging

The aqueous extract of *B. hispida* fruit showed a high nitric oxide scavenging activity, with an IC_50_ value of 85.55 ± 2.06 µg/mL, whereas standard BHT shows 42.82 ± 1.08 µg/mL, indicating its antioxidant potential (Table 3). This antioxidant protects against oxidative stress by neutralizing excess free radicals in aerobic organisms.

### 3.4. Behavioral Studies

#### Screening of LC_50_

**Figure 2:**
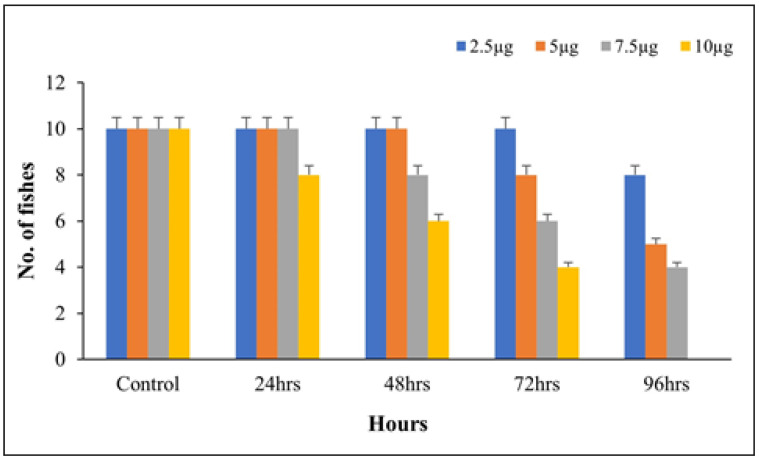
LC_50_ of aqueous extract of *B. hispida* fruit. Error bar represents the standard deviation from the mean of three independent replications (n ≥ 3).

The result monitored the aqueous extract of *B. hispida* fruit toxicity on zebrafish models for 96 h, predicting fish death rates (Figure 2). The maximum death was observed at 10 µg/mL, and 50% at 5 µg/mL, indicating a consistent dosage range of 1–3 µg/mL for stress and anxiety treatment.

### 3.5. Open Tank Test: Stress

**Figure 3:**
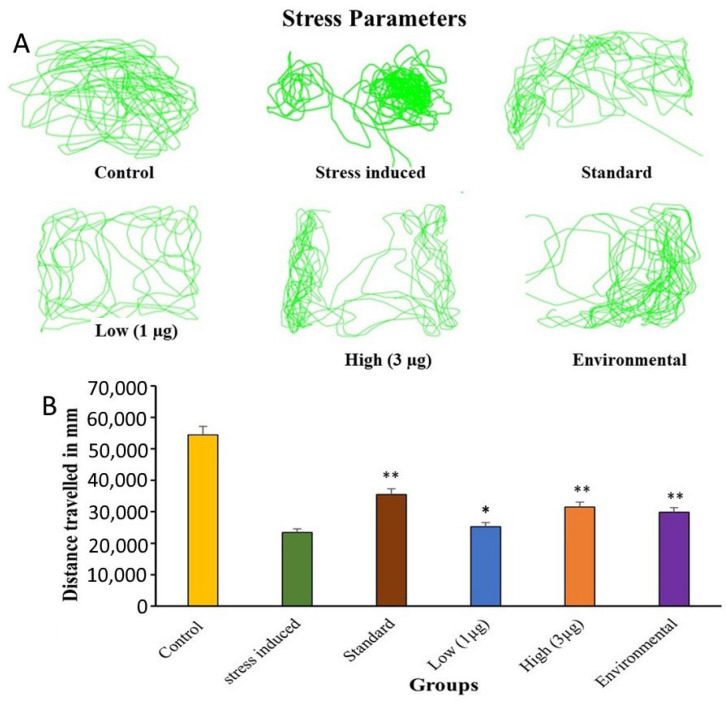
(**A**) Zebra fish stress group and aqueous extract of *B. hispida* fruit treated trajectories in open tank and (**B**) difference in open-tank test. Error bar in panel B represents the standard deviation from the mean of three independent replications (n ≥ 3). * and ** indicates the statistically significant difference at *p* ≤ 0.01 and 0.001, respectively.

The open-tank test (OTT) is a useful tool to assess stress and anxiety-related behaviors in zebrafish. This study investigated the effects of the aqueous extract of *B. hispida* fruit on stress and anxiety-related behaviors in zebrafish. For this test, a different tank was used with the dimensions L × B × H = 30 cm × 30 cm × 10 cm and the entire tank was considered for locomotion. The camera was fixed on top of the tank (top view) the trajectories were analyzed using Taxtrac software Version 2.84. Results showed that the treatment reduced frozen movements (fish will stay in one place, with only the fins and gills moving—do not swim) and improved exploration. The study also found that increasing the extract dosage caused an increase in the fish’s activity and movement, which eventually led to a decrease in stress levels. The findings suggest that the aqueous extract of *B. hispida* fruit treatment may have a significant impact on stress and anxiety-related behaviors in zebrafish (Figure 3A).

The study found that stress manipulations in adult zebrafish led to a significant reduction in locomotor activity, indicating increased stress-like behavior. This suggests that the aqueous extract of *B. hispida* fruit significantly impacts stress-related behaviors and locomotion in the OTT paradigm as shown in Figure 3B. The findings have implications for understanding how the aqueous extract of *B. hispida* fruit reduces stress-related complications.

#### 3.5.1. Open Tank Test: Anxiety

**Figure 4:**
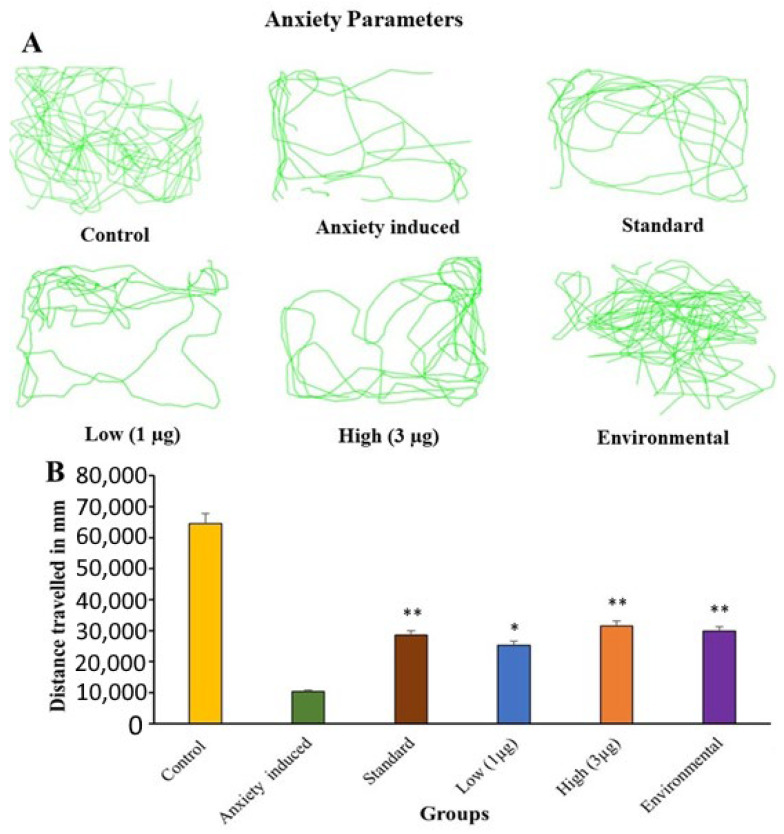
(**A**) Zebrafish anxiety group and *B. hispida*-treated group trajectories in open-tank test. (**B**) Behavioral effects of anxiety manipulation in the open-tank test. Error bar in panel B represents the standard deviation from the mean of three independent replications (n ≥ 3). * and ** indicates the statistically significant difference at *p* ≤ 0.001 and 0.0001, respectively.

The study demonstrates that anxiety manipulations in adult zebrafish can significantly impact their anxiety-related behaviors and locomotion [45]. This procedure of the experiment remains the same as stress, the fish group which has taken stress and anxiety are different. Anxiety was induced using caffeine. So, there is a difference in the behavior of stressed and anxious fish. The experimental group subjected to anxiety showed a reduction in distance traveled and locomotion activity compared to the control group. The aqueous extract of *B. hispida* fruit treatment group and environmental enrichment group showed similar locomotion activity as shown in Figure 4A. These findings suggest that the aqueous extract of *B. hispida* fruit has a significant impact on anxiety-related behaviors and locomotion in zebrafish in the OTT paradigm as demonstrated in Figure 4B. The findings highlight the potential of this assay to uncover insights into the effects of various treatments or conditions on zebrafish behavior.

#### 3.5.2. Light–Dark Test

The light–dark test is a widely used behavioral assay in zebrafish research, providing insights into stress and anxiety responses. This study uses a zebrafish model to investigate the effects of the aqueous extract of *B. hispida* fruit on stress- and anxiety-related disorders. As per the protocol mentioned in the methods, the camera was fixed on top of the tank where both the dark and light zones of the tank were visible and the video was recorded and analyzed using Taxtrack. Zebrafish are valuable models for studying neurobehavioral responses due to their genetic similarity.

#### 3.5.3. Light–Dark Test—Stress Induced and Treated

**Figure 5:**
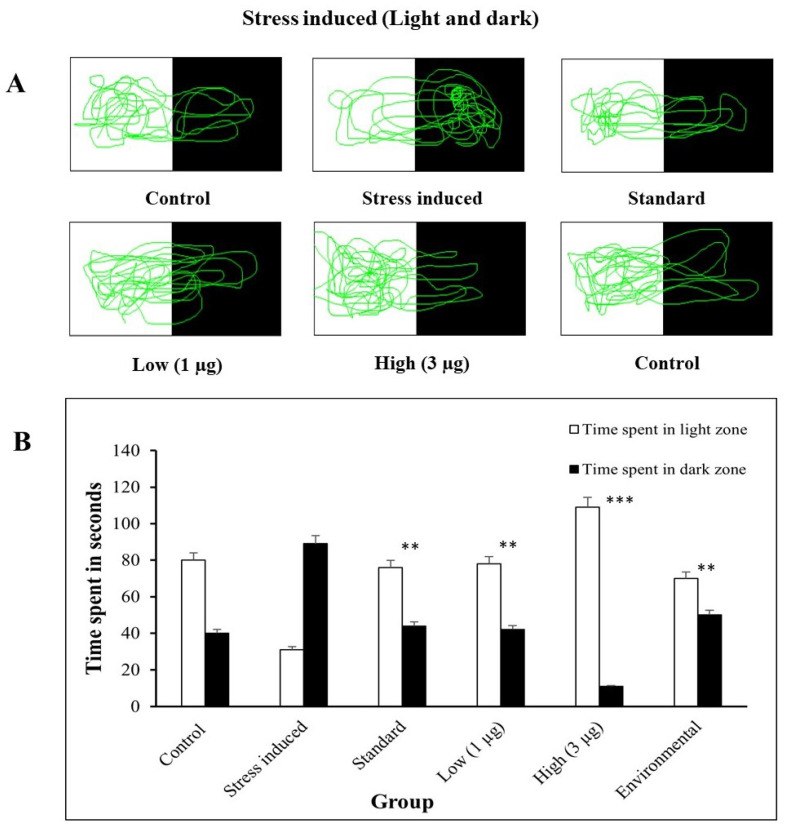
(**A**) Light–dark test trajectories in stress-induced zebrafish and light–dark test trajectories of stressed zebrafish treated with the aqueous extract of *B. hispida* fruit (low—1 µg, high—3 µg). (**B**) Comparison of zebrafish time spent in light zone/dark zone in stress-induced group and treated group. Error bar in Panel B represents the standard deviation from the mean of three independent replications (n ≥ 3). ** and *** indicates the statistically significant difference at *p* ≤ 0.001 and 0.0001, respectively.

Zebrafish behavior in a 5 min light–dark test was evaluated using the ToxTrac organism video tracking application. The results showed that stress affected the time spent in the dark side of the tank, with all groups spending more time in the dark zone. This suggests that the fish were more stressed and preferred the dark zone [46], as shown in the graph. The zebrafish trajectories after treatment show that the *B. hispida* fruit extract reduces stress by increasing time spent in the light side of the tank. The control group spent more time in the dark zone, while the standard group spent more time in the light zone. The results shown in Figure 5 suggest that all fish prefer the light zone, indicating normal stress levels.

#### 3.5.4. Light–Dark Test—Anxiety Induced and Treated

**Figure 6:**
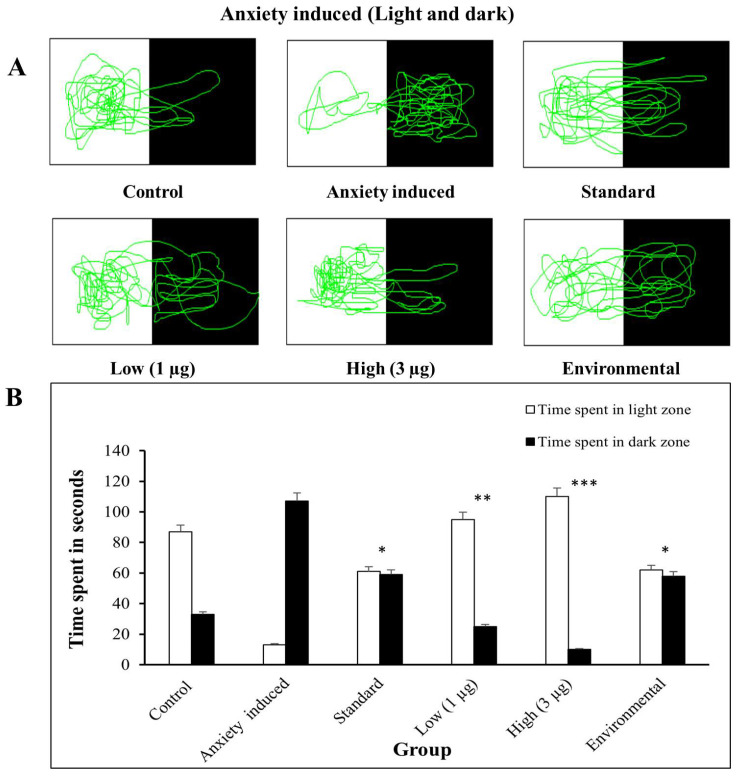
(**A**) Light–dark test trajectories of anxiety-induced zebrafish and treated zebrafish. (**B**) Comparison of zebrafish time spent in the light zone/dark zone in anxiety-induced group and group treated with aqueous extract of *B. hispida* fruit (low—1 µg, high—3 µg). Error bar in Panel B represents the standard deviation from the mean of three independent replications (n ≥ 3). *, **, and *** indicates the statistically significant difference at *p* ≤ 0.01, 0.001, and 0.0001, respectively.

The zebrafish trajectories exhibit anxiety-induced movement, with more time spent in the tank’s dark side. The experiment protocol stayed the same as for the stress test; however, the fish groups that received stress and anxiety differed. Caffeine was used to cause anxiety, so there was a difference in the behavior of stressed and anxious fish. The graph reveals that all groups spent more time in the dark zone, implying that they liked it owing to anxiety. The study reveals that anxiety may cause a preference for the dark zone. The zebrafish trajectories following treatment reveal that an aqueous extract of *B. hispida* fruit reduces anxiety, with greater time spent in the light zone, indicating that these fish have normal or reduced anxiety. The study examines the behavioral consequences of several experimental circumstances on zebrafish, including changing the levels of exposure to different chemicals. The control group spent more time in the light zone, which suggests typical behavior. The anxiety-induced groups spent more time in the dark zone, indicating anxiolytic properties. The aqueous extract of *B. hispida* fruit-treated groups showed an equal preference for both zones, indicating reduced stress- and anxiety-like behavior (Figure 6). This demonstrates the effectiveness of the light–dark test in zebrafish studies.

### 3.6. T-Maze Test

The T-maze test is a widely used behavioral assay in zebrafish research to assess cognitive and learning abilities. It involves a simple T-shaped maze, exposing zebrafish to decision-making tasks involving spatial memory and learning. The results provide insights into zebrafish’s ability to navigate and remember specific paths [47]. For example, a study involving stress and anxiety was conducted.

#### 3.6.1. T-Maze: Stress Induced and Treated

**Figure 7:**
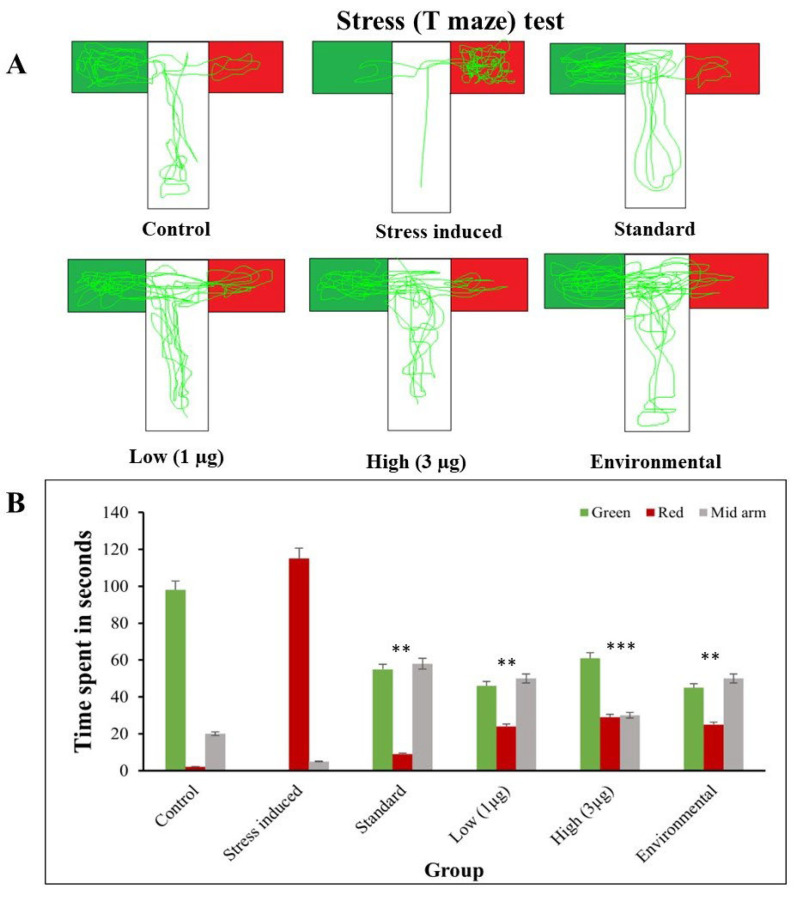
(**A**) Trajectories of stress-induced and treated zebrafish in T-maze test and (**B**) comparison of time spent in arms of T-maze when zebrafish are stressed and treated with aqueous extract of the *B. hispida* fruit. Error bar in Panel B represents the standard deviation from the mean of three independent replications (n ≥ 3). ** and *** indicates the statistically significant difference at *p* ≤ 0.001 and 0.0001, respectively.

To evaluate the memory-impairing effects of the *B. hispida* fruit aqueous extract, zebrafish behavior was measured using a T-maze test with both green and red signals (Figure 7). In the control group, spending 98 s in the green arm with food suggested adequate learning and reliable decision-making. However, the stress-induced groups demonstrated memory retention difficulties, as seen by wrong decision points and a lack of specific preference, implying a failure to remember the proper path. The study also looked at how the aqueous extract of *B. hispida* fruit affected memory in zebrafish. The results showed that the control group spent more time in the green arm, which included food. In contrast, groups given an aqueous extract of *B. hispida* fruit and those subjected to environmental enrichment had improved cognitive ability, notably in spatial memory and learning. *B. hispida* treated groups spent more time in the green arm, implying that stress-induced memory impairments could be mitigated. Notably, these treated groups consistently preferred the proper arm of the T-maze, indicating enhanced cognitive abilities, as seen in Figure 7A,B. The results of this study reveal that an aqueous extract of *B. hispida* fruit may reduce stress-induced memory impairment in zebrafish, resulting in better cognitive capacities, notably spatial memory and learning. These findings shed light on the possible cognitive benefits of *B. hispida* and its extract, paving the door for further studies on natural chemicals and their effects on memory.

#### 3.6.2. T-Maze: Anxiety Induced and Treated

**Figure 8:**
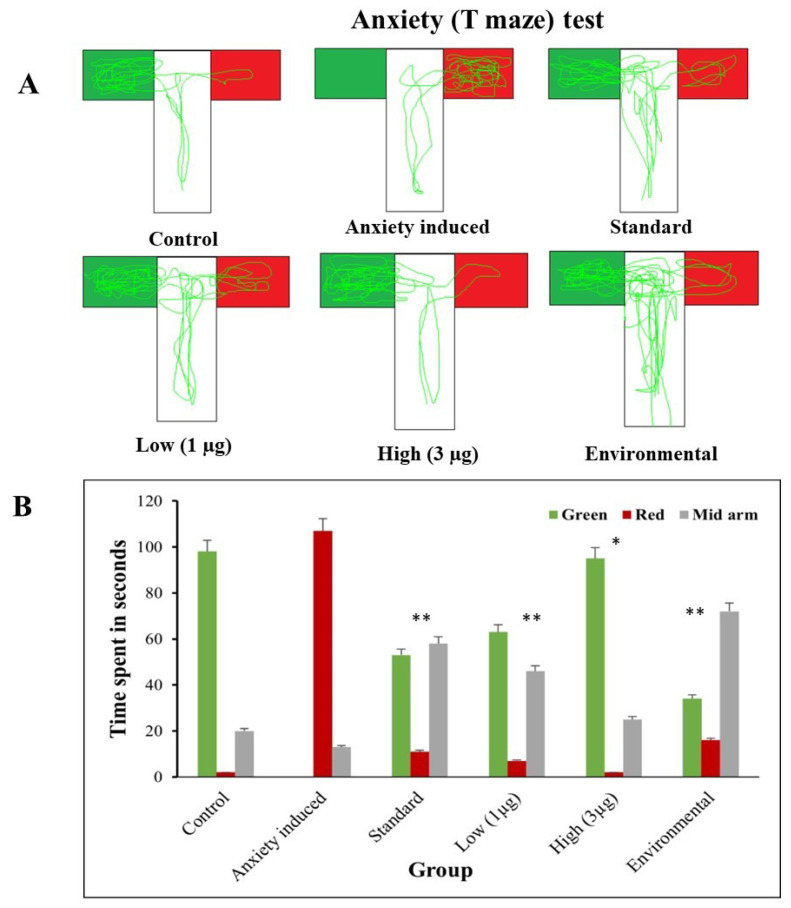
(**A**) Trajectories of anxiety-induced and treated zebrafish in T-maze. (**B**) Comparison of time spent in arms of T-maze when zebrafish were induced with anxiety and treated with aqueous extract of *B. hispida* fruit. Error bar in Panel B represents the standard deviation from the mean of three independent replications (n ≥ 3). * and ** indicates the statistically significant difference at *p* ≤ 0.01 and 0.001, respectively.

The study aims to evaluate the memory-altering effects of an aqueous extract of *B. hispida* fruit in zebrafish, comparing normal and anxiety-induced groups. Figure 8 shows that the time spent in the food-containing green arm decreased in the anxiety-induced groups, indicating a reduction in memory retention. These groups had wrong choice points and no clear preference, indicating a failure to remember the correct path. The control group, *B. hispida*-treated groups, and environmental enrichment groups were all tested for cognitive function. Figure 8 shows that the control group spent more time in the food-containing green arm. In contrast, both the *B. hispida*-treated and the environmentally enriched groups had improved cognitive ability, particularly spatial memory and learning. The increased time spent in the food-containing green arm in the *B. hispida*-treated groups indicates that anxiety-induced effects on cognitive function may be mitigated. The study’s findings show that an aqueous extract of *B. hispida* fruit may help zebrafish improve their cognitive abilities, notably spatial memory and learning. These findings provide useful insights for future research into the effects of natural chemicals on cognitive function, as well as their potential applicability in understanding neurological illnesses and environmental factors influencing cognitive performance.

### 3.7. Biochemical Parameters

#### 3.7.1. Estimation of Superoxide Dismutase (SOD) and Catalase (CAT) Activities

**Figure 9:**
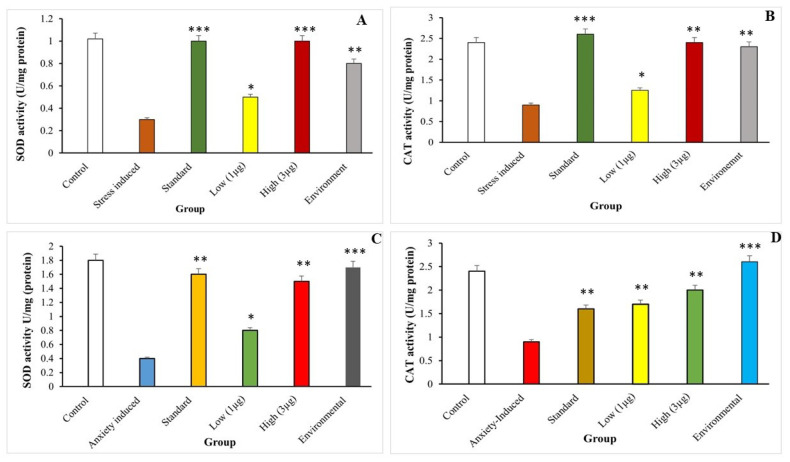
SOD and CAT activity of stress and anxiety in zebrafish. (**A**) SOD activity of stress group, (**B**) CAT activity of stress group, (**C**) SOD activity of anxiety group, and (**D**) CAT activity of anxiety group. Error bar represents the standard deviation from the mean of three independent replications (n ≥ 3). *, **, and *** indicates the statistically significant difference at *p* ≤ 0.01, 0.001, and 0.0001, respectively.

The study found that stress-induced brain samples showed a decrease in SOD activity compared to control cells, but increased in a dose-dependent manner in the Aqueous extract of *B. hispida* fruit. The extract significantly increased SOD activity, while catalase (CAT) activity also increased significantly. aqueous extract of *B. hispida* fruit had a more uniform distribution and stronger antioxidant activity (Figure 9).

The study assessed the effects of the aqueous extract of *B. hispida* fruit on stress-induced zebrafish’s SOD and CAT, (Figure 9A,B). SOD converts free radicals into oxygen and hydrogen peroxide, while CAT converts hydrogen peroxide into water and oxygen. The antioxidant effect of the aqueous extract of *B. hispida* fruit was indirectly estimated. The effects of the aqueous extract of *B. hispida* fruit on the anxiety-induced zebrafish’s SOD and CAT (Figure 9C,D) focus on the antioxidant effects of SOD, which converts free radicals into oxygen and hydrogen peroxide, and the catalase activity, which indirectly reduces anxiety.

#### 3.7.2. Acetylcholinesterase Activity

**Figure 10:**
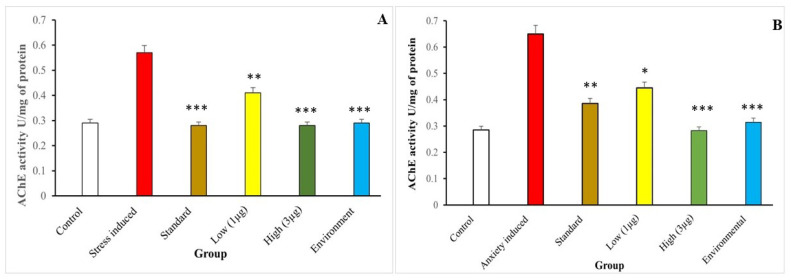
Aqueous extract of *B. hispida* fruit (1, 2, and 3 µg/mL) exhibited an anti-AChE effect and improved the antioxidant status in the stress-induced (**A**) and anxiety-induced (**B**) zebrafish brain. Error bar represents the standard deviation from the mean of three independent replications (n ≥ 3). *, **, and *** indicates the statistically significant difference at *p* ≤ 0.01, 0.001, and 0.0001, respectively.

Flavonoid glycoside can improve the dyskinesia recovery rate in zebrafish, a common complication of neurodegenerative diseases. Acetylcholinesterase inhibitors can improve cognitive function and reduce falls in neurodegenerative patients. The aqueous extract of *B. hispida* fruit, with 90% inhibitory activity against stress (Figure 10A) and anxiety (Figure 10B), may be a potential natural product for treating neurological diseases like stress and anxiety (Figure 10). The extract contains homogalacturonan, phenolic compounds, and flavonoids, suggesting more effectiveness and less toxicity.

## 4. Discussion

Research on medicinal plants can lead to the identification and development of new treatments for stress and anxiety disorders [48]. To the best of our knowledge, this is the first study to show that *B. hispida* fruit extract prevents zebrafish stress and anxiety reactions. Plant sources and isolated phytochemicals have a wide range of pharmacological effects on brain-related disorders such as anxiety, depression, stress, and cognitive deficiencies, sparking interest in the development of therapeutic drugs. Polyphenolics and flavonoid compounds are found in almost every plant, including fruits, flowers, seeds, and vegetables. In rodent models, a variety of important flavonoids derived from fruits and plants have shown promising preclinical effects on emotional disorders such as stress and anxiety [49]. Zebrafish, a non-human animal, have advantages in modeling brain illnesses since they share behavioral features, genetic factors, and pharmacological sensitivity with rat models. This model is widely used for studying stress-induced brain changes [50]. The aqueous extract of *B. hispida* fruit, a fruit vegetable rich in nutritious components, has a wide range of functional properties. References [51,52] suggest that the aqueous extract of *B. hispida* fruit compounds may have therapeutic and pharmaceutical applications, including antioxidant, anti-ulcer, anti-inflammatory, ACE inhibitory, anti-obesity, and anti-compulsive properties. Knowing the foregoing context, the current study focused on the treatment of stress and anxiety with an aqueous extract of *B. hispida* fruit.

The aqueous extract of *B. hispida* fruit is an edible plant having therapeutic qualities thanks to its phytochemicals. This study discovered large yields the aqueous extract of *B. hispida* fruit, showing its solubility in water. The extracts contained phenolic components, as confirmed by phytochemical analysis, which is consistent with the findings of Phumat [53], who found phenolic compounds as well as other substances such as quercetin, rutin, astilbin, catechin, naringenin, and hispidulin [54].

The antioxidant properties of the aqueous extract of *B. hispida* fruit have been assessed using established techniques to determine the various mechanisms of action. DPPH, H_2_O_2_, and Nitric oxide scavenging techniques which are routinely employed to test the antioxidant activity of the substances acting as scavengers of O_2_−• and •OH radicals, and these findings are similar to those [55,56,57].

Behavioral parameters in the current study have shown that there is a restricted is frozen movement behaviors when the fish were induced by stress and anxiety. There was a gradual increase in the locomotory activities when an aqueous extract of *B. hispida* fruit was given as a treatment, supporting these results. Ambikar and Mohanta [58] described locomotor activity in a mouse model. A baseline activity score was acquired after five minutes of use in the actophotometer. Eleven groups of animals were then created and given an aqueous extract of *B. hispida* fruit treatment. Sixty minutes following the dose, the mice were once more put in the actophotometer to record the previously mentioned activity score.

Puri, et al., [59] reported that zebrafish and humans share a similar physiology, hence zebrafish have been utilized as models for studying anxiety. Despite being well understood, the pathophysiology of anxiety has been extensively studied in zebrafish. Light–dark transitions (unpleasant circumstances) alter zebrafish locomotor activity, with a shift from light to obscurity resulting in hyper movement due to increased pressure and pain [60]. The aqueous extract of *B. hispida* fruit water concentrations of 1.5 and 3.5 mg/L had a significant effect on zebrafish when compared to the control group. The aqueous extract of *B. hispida* fruit has long been regarded as a plant of interest due to its antistress properties and ability to treat mental health concerns [61,62].

The results were compared to other studies that focused on rodents or other animals. This study suggests that the aqueous extract of *B. hispida* fruit can reduce stress and anxiety in both behavioral and biochemical parameters. This could be due to the phenolic chemicals found in *B. hispida* fruit extract, or it could be related to its synergistic efficacy in lowering stress and anxiety in zebrafish models. Additional biochemical findings support the action of the aqueous extract of *B. hispida* fruit. This discovery is consistent with the increasing demand for natural therapeutic adjuvants, such as herbal medicines in the Ayurvedic medical system. Furthermore, evidence-based research and in-depth investigations may decrease the progression of neurological diseases.

## 5. Conclusions

The behavioral measure obtained after stress exposure confirms the neuropharmacological effect of the aqueous extract of *B. hispida* fruit on stress-induced anxiety. With its established capacity to reduce the effects of acute stress, aqueous extract of *B. hispida* fruit emerges as a promising therapeutic option for stress- and anxiety-related mental diseases. However, despite its noteworthy efficacy and safety, the aqueous extract of *B. hispida* fruit has not received regulatory approval as a pharmaceutical treatment. Further study and a regulatory processes are required to confirm its status as an approved medicinal treatment.

## Figures and Tables

**Table 1:** Procedure of the stress protocol in zebrafish.

Days	Stresses
Monday	9:00 a.m. Chasing
	1:00 p.m. Cooling
Tuesday	11:00 a.m. Social isolation
	3:00 p.m. Crowding
Wednesday	10:00 a.m. Restraint stress
	2:00 p.m. Low water level
Thursday	12:00 p.m. Net stress
	4:00 p.m. Tank water replacement

**Table 2:** Phytochemical screening of aqueous extract of *B. hispida* fruit.

Tests	*Benincasa hispida* Fruit (Aqueous Extract)
Phytosterols	+
Saponins	+
Alkaloids	−
Carbohydrates	+
Anthroquinone	+
Flavanoids	−
Phenolic compounds & Tannins	+
Steroids	−
Proteins	+
Fixed oils & Fatty acid	+

+ Positive; − Negative.

**Table 3:** Antioxidant activity of *B. hispida* fruit.

Assay	Standards (µg/mL)	Aqueous Extract of *B. hispida* Fruit (µg/mL)
DPPH	40.41 ± 1.6 (BHT)	54.56 ± 1.08
H_2_O_2_	44.37 ± 1.9 (AA)	67.53 ± 0.05
NO	42.82 ± 1.08 (BHT)	84.55 ± 2.05

## Data Availability

Data are contained within the article.
